# Hydration-induced modulation of aromaticity and reactivity in anthocyanidins: a quantum mechanical study†

**DOI:** 10.1039/d5ra05334j

**Published:** 2026-01-22

**Authors:** Ajay Shankar

**Affiliations:** a Department of Chemistry, Indira Gandhi National Tribal University Amarkantak Madhya Pradesh-484887 India ashankar@igntu.ac.in

## Abstract

This study explores the hydration effects on aromaticity, orbital delocalization, and local and global reactivity descriptors of anthocyanidins. Using the Harmonic Oscillator Model of Aromaticity (HOMA) and *para*-delocalization index (PDI), the quantitative assessment of the aromaticity of the three rings in anthocyanidins is performed. It revealed that the hydration generally increases their aromaticity, though the extent of change varies across different rings in the skeletal structure. The HOMA and PDI indices show consistent trends in aromaticity, with ring B exhibiting the highest aromaticity, followed by fused rings, *i.e.*, ring A and ring C. The study also highlights the role of orbital delocalization indices (ODI) in understanding the hydration effects on the electronic structure of anthocyanidins. A significant increase in the delocalization of electrons in the HOMO post-hydration is observed. The global reactivity descriptors computed under conceptual density functional theory (CDFT) show that the hydration of anthocyanidins affects their reactivity towards electron-rich species. The acidic forms of anthocyanidins are characterized as superelectrophiles whose nature is retained while the electrophilic strength decreases slightly upon hydration. The decrease in global chemical hardness (*η*), increase in global nucleophilicity (NI), and changes in vertical ionization potential (VIP) and vertical electron affinity (VEA) indicate the differences in reactivity arising from the hydration of anhydrous forms of anthocyanidins. This should have implications for their antioxidant properties and potential applications in food packaging and smart sensors for quality monitoring of protein-rich foods such as meat and fish, which are perishable in nature. The atomic level attribution over the 2D chemical structures created a heatmap, which assisted the local reactivity analysis using condensed dual descriptors (CDDs). The heatmap overlapped with 2D chemical structures highlighted the specific sites, mostly in the fused ring C, suggesting it to be more favourable for nucleophilic attack after hydration. This observation reinforces the alteration in reactivity as predicted from the several global descriptors of the anthocyanidin molecules. Overall, this comprehensive analysis provides a deeper understanding of the hydration effects on the chemical behaviour of anthocyanidins, offering valuable insights into their applications.

## Abstract 2

This study explores the hydration effects on aromaticity, orbital delocalization, and local and global reactivity descriptors of anthocyanidins, with relevance to food packaging and its monitoring.

## Graphical abstract

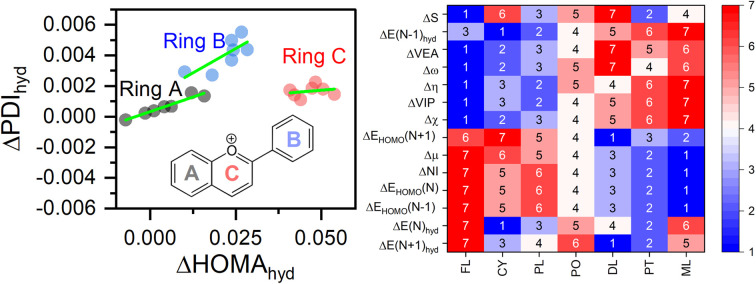

## 1 Introduction

Anthocyanins are a diverse group of water-soluble pigments found in a wide array of fruits, vegetables, and flowers. These compounds are well known for their vibrant colours and potential health benefits, including antioxidant and anti-inflammatory properties. Anthocyanins are glycosylated forms of anthocyanidins. They consist of an anthocyanidin core molecule attached to one or more sugar molecules. Structurally, anthocyanins consist of a flavonoid backbone with various substitutions that influence their colour and reactivity.^1^ The presence of hydroxyl groups, methoxy groups, and other substituents modulates the electronic properties of anthocyanidins and, consequently, their stability and reactivity. There are 27 anthocyanidins and approximately 702 anthocyanins identified so far that exist in nature. It is interesting to note that a total of six anthocyanidins, *viz.*, malvidin (MV), cyanidin (CY), pelargonidin (PL), peonidin (PO), petunidin (PT) and delphinidin (DL) mainly constitute the anthocyanin intake in the diets of humans and this accounts for more than 90% of known anthocyanins. The anthocyanidins seldom exist alone in their natural occurrences.^2–5^ The glycosylated form of anthocyanidins known as anthocyanins extracted from a variety of plants, including purple sweet potato,^6^ torch ginger,^7^ black bean seed coat,^8^ red cabbage,^9^ blueberry,^10^ black rice bran,^11^ black plum,^12^ roselle,^13^ black carrot^14,15^ and so on^16–22^ have been explored for the development of active and smart food packaging films. However, the high cost associated with the extraction, separation and isolation of anthocyanidins currently limits their potential for cost-effective applications. This current limitation encourages the use of quantum mechanical methods for a better understanding towards their applications. Anthocyanins may contain around 100 atoms, depending on their glycosylation and acylation patterns whereas, simpler anthocyanidins alone have around 40 atoms. Thus, in a typical computational study, anthocyanidins shall serve as model systems to understand the flavylium ion core-based applications of anthocyanins. The Density Functional Theory (DFT) is preferred over other modern quantum mechanical methods due to its favourable balance between accuracy and computational efficiency.^23,24^ Unlike post-Hartree–Fock approaches, which can be computationally demanding for large systems, DFT provides a robust approximation of electronic structure through functionals that are computationally more manageable. This efficiency, combined with the ability to handle complex molecular systems and predict electronic properties with reasonable accuracy, makes DFT a widely used and practical choice for studying molecular and periodic systems.

One of the critical aspects of anthocyanin and anthocyanidin chemistry is the impact of hydration on their electronic structure and aromaticity. Hydration, which can occur under physiological conditions or in moist and aqueous environments, affects the delocalization of electrons within the aromatic rings of anthocyanidins. This delocalization is crucial for understanding their chemical behaviour, stability, and colour changes. In particular, the aromaticity of these rings, which is a measure of the delocalized electron cloud, can significantly influence the reactivity of molecules and their interaction with other chemical species. These are important aspects when they are being considered and employed as in food packaging and smart sensors for food quality monitoring,^25–31^ apart from their other applications such as functional foods, nutraceuticals, cosmetics, and pharmaceutical products.^32–34^ Globally, non-vegetarian diets are far more common, with more than 90% of the world's population consuming some form of meat or fish. Such type of foods typically possesses high protein content and make them play an important role in the human diet. The decomposition of protein by its oxidation in meat mostly produces reactive oxygen species and detectable amounts of volatile organic amines, and gaseous ammonia, which are basic in nature. The pH-responsive nature of anthocyanins displayed by their significant colour changes observable from the naked eye enables them as a promising candidate in the near future for food packaging and monitoring of shelf life.^35,36^

The hydration effect on aromaticity has been studied in various systems, but the specific changes in anthocyanidins, particularly in their different rings, remain a subject of significant interest. The current study investigates how hydration alters the aromaticity of aromatic rings in the seven anthocyanidins. Furthermore, it explores the changes in orbital delocalization indices, specifically focusing on the highest occupied molecular orbital (HOMO) and the lowest unoccupied molecular orbital (LUMO). There are several ways to characterize aromaticity, of which the Nucleus-Independent Chemical Shift (NICS) index is often used.^37,38^ It measures the magnetic shielding at or near the geometric centre of a ring and serves as one of the reliable indicators of aromaticity or anti-aromaticity. Aromatic systems generally exhibit negative NICS values due to the induced diatropic ring current, while anti-aromatic systems display positive values from paratropic currents. The NICS indices directly probe the magnetic response, offering a complementary perspective on aromaticity when the same is being determined using the harmonic oscillator model of aromaticity (HOMA) and *para*-delocalization index (PDI).

This study aims to bridge the gap between structural changes and reactivity by providing a detailed analysis of how hydration impacts aromaticity, orbital delocalization, and global chemical properties of anthocyanidins. The understanding of these effects is crucial for the development of anthocyanin- and anthocyanidin-based sensors for assessing the freshness of food and other applications where hydration conditions can alter their chemical behaviour. The choice of acidic forms of anthocyanidins is justified by their stability^39^ and emerging applications in the monitoring of the quality of packaged non-vegetarian food.^21,22^

**Fig. 1:**
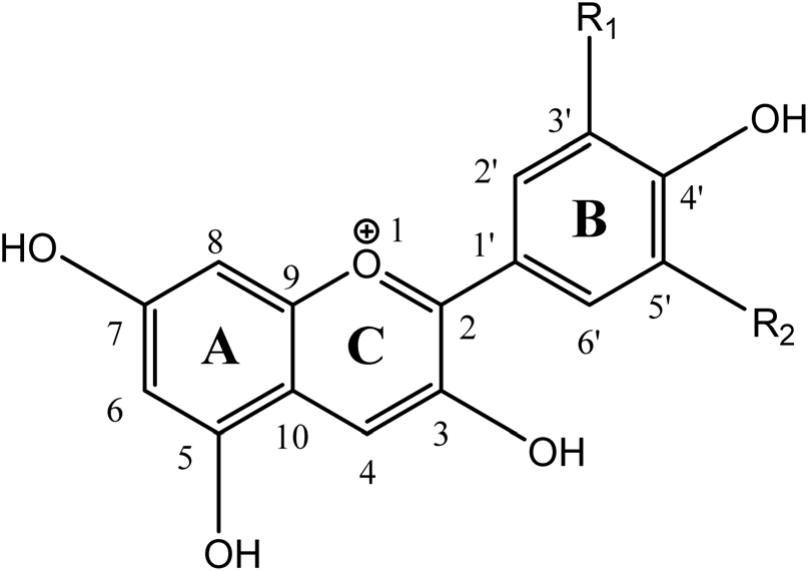
Structural detail of the seven anthocyanidins. The functional groups attached to the B and C rings for each anthocyanidin are taken as mentioned here. MV, R_1_ = –OMe, R_2_ = –OMe; CY, R_1_ = –OH, R_2_ = –H; PL, R_1_ = –H, R_2_ = –H; PO, R_1_ = –OMe, R_2_ = –H; PT, R_1_ = –OMe, R_2_ = –OH; DL, R_1_ = –OH, R_2_ = –OH.

A ring-by-ring qualitative indexation (as shown in Fig. 1) makes them very useful for comparison of the aromaticity.

## 2 Theoretical calculation details

The density functional theory was used for all the quantum mechanical calculations of anthocyanidins. Their acidic forms are considered in all these calculations. In a typical procedure, the optimization of the ionized molecular structure of anthocyanidin (without any counterion) is carried out using the Becke three-parameter hybrid functional. The basis set 6-311G++(d,p) is employed in the calculations,^40–43^ which have been previously tested on anthocyanins.^44,45^ Two sets of geometry optimization on all anthocyanidins are carried out to determine the ground state, one in the gaseous phase and the other in the aqueous phase. Grimme's empirical dispersion correction was also considered with Becke–Johnson damping,^46^ which provides an opportunity to study weak non-bonding interactions. A pruned ultrafine integration grid was employed with a tight self-consistent field procedure. In addition, an extra SCF step was kept to overcome the non-convergence of the first SCF step, if any. For the optimization in the aqueous phase, the polarizable consistent field (PCM) using integral equation formalism, *i.e.*, the IEFPCM method, was used as a self-consistent reaction field method.^47^ The DFT calculations are carried out using the Gaussian suite of programs.^48^ The conceptual density functional theory (CDFT) calculations are performed in the Multiwfn program, version 3.8.^49,50^ The Hirshfeld analysis requested atomic densities by using built-in sphericalized atomic densities in free states, which were then used for obtaining orbital delocalization indices of all atoms in a typical anthocyanidin molecule. It was done separately for two different phases.

The computations under the density functional theory DFT yielded the energies of the molecules under study. They act as the main ingredient for the determination of global reactivity descriptors of the three-dimensional molecular structure of interest. The *E*(*N*), *E*(*N* + 1), *E*(*N* − 1), VEA, VIP, *η*, *S*, *χ*, *µ*, NI, and *ω* represent the energy of molecule, energy of molecule with extra electron, energy of molecule with one electron less, vertical electron affinity, vertical ionization potential, global chemical hardness, global chemical softness, Mulliken electronegativity, chemical potential, global nucleophilicity index and global electrophilicity index, respectively. The determination of these global quantum descriptors does not require any data from a reference molecule except for, global nucleophilicity index. The *N* represents the total number of electrons of the molecule under study, *i.e.*, positively charged anthocyanidin molecules considered in this work. The *N* + 1 and *N* − 1 represent the hypothetical situations where these molecules have gained and lost one electron, respectively. The mathematical calculation steps describing the determination of several global parameters are summarized below.

The global hardness is given as^51^

1 *η* = VIP − VEA

Thus, the global softness can be written as2
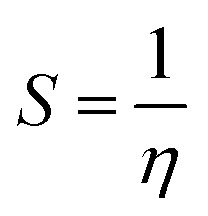


The DFT description of Mulliken electronegativity can be written as^52^3
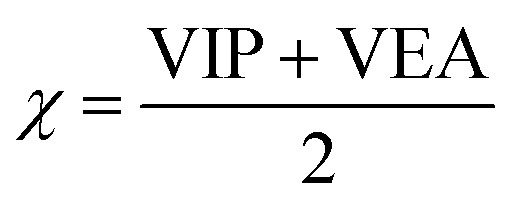


The chemical potential is the opposite of Mulliken electronegativity.

4 *µ* = −*χ*

The global electrophilicity index is calculated as^53^5
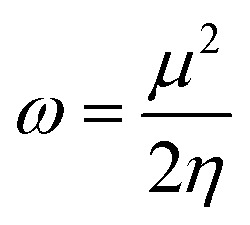


The global nucleophilicity index is defined as^54–56^

6 NI = *E*_HOMO_(Nu) − *E*_HOMO_(TCE)

The energy of HOMOs of different molecules is involved in the calculation, *i.e.*, *E*_HOMO_. The Nu denotes nucleophile, which is the same as the molecule of interest. The TCE is tetracyanoethylene, which is generally taken as the reference for the calculation of this global parameter. It has been calculated in this study using the same level of theory in DFT as that of the nucleophile. The local reactivity analysis was assisted by an explainable SMILES representation, also known as XSMILES, as shown recently in some of the instances.^57,58^

## 3 Results and discussion

To provide a conceptual reference point for understanding electronic and hydration-related trends, the flavylium cation (FL) was included in the list of anthocyanidins for comparative analyses. Unlike anthocyanidins, the FL cation lacks hydroxyl or methoxy substituents on the aromatic rings, making it a structurally minimal core. This structural simplicity allows it to serve as a baseline for assessing the influence of different functional groups present in the anthocyanidins. Notably, the effects of hydration on the FL cation follow trends similar to those observed for the anthocyanidins. For these reasons, FL has been discussed along with anthocyanidins and supports the comparative analysis presented in this study.

### 3.1 Inter- and intra-ring hydration effects on aromatic rings

The aromaticity of the rings in anthocyanidins is anticipated to be influenced by hydration effects. In this work, the delocalization of electrons over the plane of rings of anthocyanidins in their acidic forms is measured using HOMA and PDI.^59–61^ The former provides structure-based criteria, while the latter uses electronic information for measuring the aromaticity of rings. The HOMA index is one of the structure-based measures to know about the aromaticity in different parts of the molecules. It can be written as^59,60^7
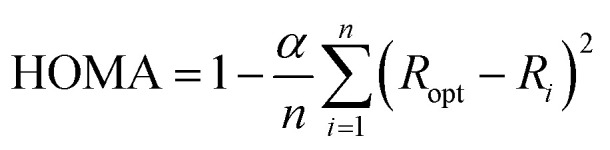


The *n* represents the number of bonds being analysed, while the constant is an empirical value chosen to set the HOMA index to 0 or 1. The term *R*_*i*_ denotes the bond length of the *i*th bond in the system.

For the measurement of this index, the complete aromatic systems are chosen, and their bond lengths are taken as references for comparison in the test systems.^49,50^ The two extreme cases concerning this measure are as follows. When HOMA equals 1, it indicates the complete aromatic nature in test compounds as a result of a match of bond length with the reference compound. If the HOMA value equals 0, it signifies a completely non-aromatic nature. The calculation of HOMA is sensitive to the choice of reference bond lengths. The aromaticity of the three rings, as revealed by their HOMA indices in anthocyanidins, is differentiable. For any particular anthocyanidin species, it can be seen that the extent of aromaticity in the three rings of interest, as can be visualized from HOMA indices, follows the order B > A > C. For the C–O bond in ring C, the bond length of carbon monoxide is taken as a reference in the calculation of HOMA indices. For C–C bonds, it is the bonds of a benzene ring which serve as a reference. A numerical difference of *c.a.* 2% is observed for ring B and ring A, for which HOMA is always higher for the former ring in the gaseous phase of the studied molecules. This is due to the different skeletal attachments of benzene rings for ring A and ring B. The presence of oxygen as a heteroatom causes a less efficient delocalization of electrons on the ring C, which leads to its lowest HOMA indices amongst all three rings of anthocyanidins. This qualitative understanding of the order of aromaticity of anthocyanidins remains the same in the aqueous phase also (see Fig. S1a and b). The difference between the HOMA indices of both of the fused rings, *i.e.*, ring A and ring C, in any particular phase may be regarded as ΔHOMA_A–C_. It decreases for each of the studied anthocyanidins after solvation. A similar effect for ring C and ring B is observed for their HOMA indices in the plot of ΔHOMA_B–C_ (see Fig. S1c and d). A larger increase in HOMA indices for ring C is the primary cause behind the variation in ΔHOMA_A–C_ and ΔHOMA_B–C_ indices of anthocyanidins. The similar observations for the flavylium cation (as that of anthocyanidin molecules), in both phases, assert that it is the inherent nature of constituent rings in these compounds which leads to this generalized trend of aromaticity. The effect of the presence of different substituents on the ring B in these anthocyanidin molecules is visible in both phases. The effect of hydration on ΔHOMA_B–C_ index may be related to the presence of the methoxy group on ring B in the core skeletons of MV, PO and PT. In the absence of a substituent on the core skeleton, *i.e.*, in FL, the effect of hydration on ΔHOMA_A–C_ and ΔHOMA_B–C_ indices is largest. A slight dependence of π-electron cloud delocalization on the substituents can be observed from the HOMA index bar charts.

Like HOMA, the PDI also offers the advantage of using local criteria for each of the rings in assessing the aromaticity of polycyclic compounds. The quantum theory of atoms in molecules provides an electronic-based delocalization index, *δ*(*x*, *y*) between the two atoms *x* and *y*. The average of these delocalization indices for *para*-carbons in a six-membered ring is regarded as PDI whose development and validity have been detailed elsewhere.^61^ In Fig. S1e and f, it can be seen that the order of inter-ring aromaticity as predicted by PDI for the three rings is preserved fully, *i.e.*, B > A > C. The mutual differences among the three rings before and after hydration are negligible, as can be seen from the height of the bars.

**Fig. 2:**
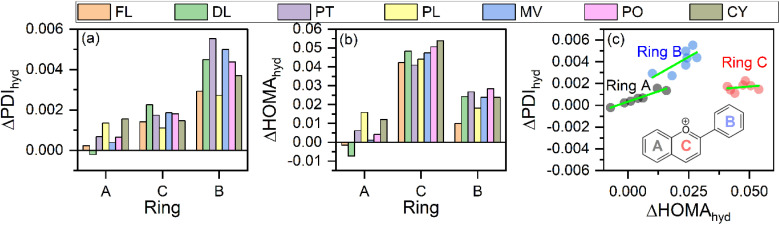
The hydration effects on the three rings of anthocyanidins as predicted by their change in (a) PDI indices, and (b) HOMA indices. (c) The correspondence between ΔPDI_hyd_ and ΔHOMA_hyd_ for the aromatic rings.

Fig. 2a and b show the effect of hydration for each ring in anthocyanidin, called here an intra-ring effect. It is calculated by subtracting the HOMA value of the ring in the gaseous phase from that of the value in the aqueous phase. This method is followed to observe the effect of hydration on all other indices, descriptors, energies and so on in the analysis of results in this work. The hydration causes both delocalization indices to increase for all the rings in anthocyanidins, including flavylium cation. The extent of increase in aromaticity post hydration, as calculated from PDI, shows the trend which can be written for rings as ΔPDI_ring A_ < ΔPDI_ring C_ < ΔPDI_ring B_ for all the anthocyanidins. However, the calculated HOMA indices for these rings show a different order. For ΔHOMA, the increase in aromaticity is largest for ring C, followed by ring B and ring A for all the anthocyanidins. Despite the differences in the overall order of the three rings for their aromaticity, as revealed from PDI and HOMA, the change in intra-ring aromaticity for all rings indicates the same trend after hydration in anthocyanidins, *i.e.*, they increase. To see the coherence between the predicted change in aromaticity after hydration by HOMA and PDI, their differences are plotted in Fig. 2c. For all of the rings in the anthocyanidins, the indices of aromaticity increase post-solvation as perceived from a linear fit on the corresponding data of three rings. The slope of linear fits on the plotted values of the rings decreases in the sequence for ring B, ring A and ring C. The differences in the prediction regarding the change in aromaticity after hydration may be due to the inherent approach followed by HOMA and PDI during their calculation. The HOMA index calculation mainly focuses on the information of bond distances, which relates to the spatial presence of atoms. The choice of reference bond distances may alter the observed trend for the rings of anthocyanidins under the HOMA index calculation. As compared to it, the PDIs utilize the integrated exchange–correlation density over the atomic basins defined under AIM theory.^62,63^ So, it provides the number of electrons delocalized and shared between the two atoms. Thus, HOMA may not capture all nuances of aromaticity in more complex or polycyclic systems, and it may be limited to simple and smaller systems. On the other hand, PDI seems more comprehensive in assessing the delocalization of electrons, providing a detailed view of aromaticity along with the flexibility arising from the focus on electronic structure. Thus, a firm background of the method employed behind the PDI calculation is perceived as compared to that of the HOMA index.

The main limitation of PDI is that it is primarily suitable for studying structural perturbations in the six-membered rings and is less reliable for molecular systems with significant out-of-plane distortions of the ring of interest within the molecule. However, neither of these limitations applies to the studied molecules in both the gaseous and aqueous phases as all anthocyanidin rings are six-membered and exhibit nearly planar structure for each of the constituent rings.

### 3.2 Effect of hydration on orbital delocalization indices

The hydration effects on the electronic structure are better understood by orbital composition analysis. Under this analysis, there are many approaches, amongst which the orbital delocalization index (ODI) is mostly preferred due to its advantages.^64^ The ODI for *i*th orbital is calculated using the following equation8
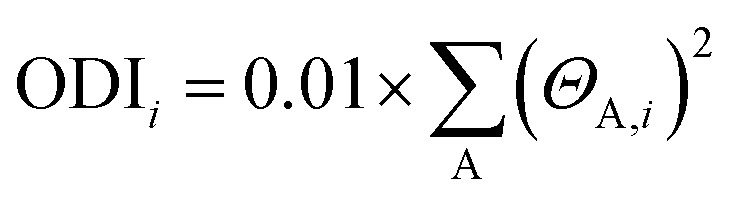


**Fig. 3:**
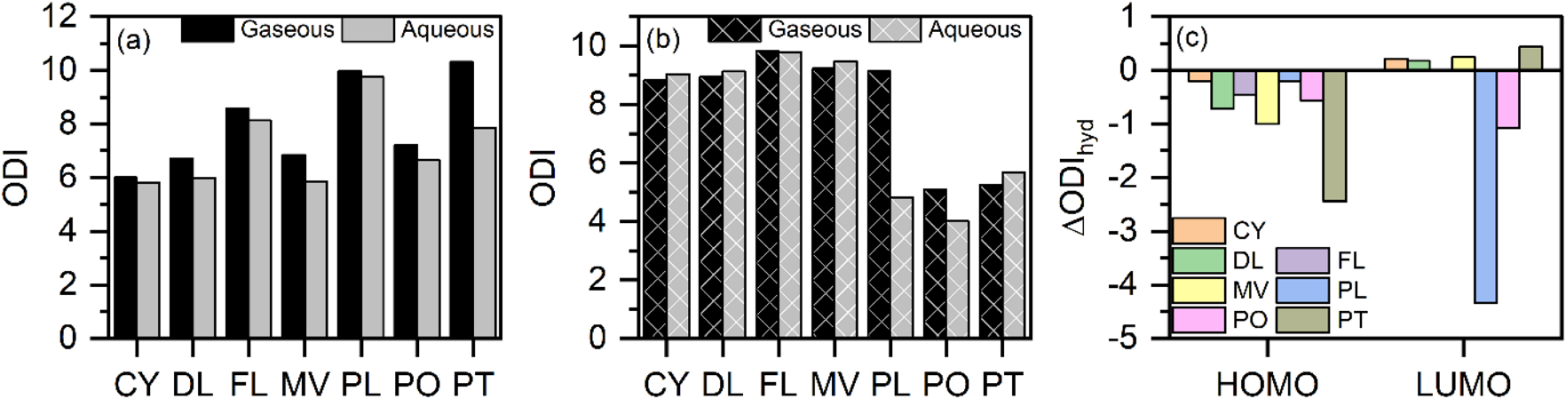
The orbital delocalization indices in gaseous and aqueous phases for (a) HOMO, and (b) LUMO of anthocyanidins. (c) The effect of hydration on ODI for the frontier orbitals of anthocyanidins.

The composition of atom A in orbital is given by *Θ*_A,*i*_. The Hirshfeld method is used to calculate the ODI of all the occupied and virtual orbitals of anthocyanidins. The delocalization index gives the composition of various atoms in the calculated orbital of the molecule under study. It acts as an indicator for the extent of delocalization; the lower the ODI value higher the orbital delocalization, and *vice versa*. From Fig. 3a, it can be seen that the HOMO of all anthocyanidins has their ODI values higher in the gaseous phase of the molecules as compared to those in their aqueous phase. This observation supports the findings drawn from the analysis of ΔHOMA_hyd_ and ΔPDI_hyd_. However, for LUMO in Fig. 3b, we do not observe any particular trend of hydration on the delocalization indices of the orbitals. The increase in the extent of delocalization of electrons is characterized by ODI values of HOMO in the anthocyanidins. The negative values of ΔODI_hyd_ shown in Fig. 3c clarify the above-discussed view on the extent of delocalization as understood from frontier orbitals. The 3D isosurface plots of the frontier orbitals are shown in Fig. S2–S8 for the studied anthocyanidins in gaseous and aqueous phases, which may also be referred. The red and blue colours represent the positive and negative parts of the wavefunction, respectively. An isovalue of 0.02 is used to assist in the visualization of changes in MOs upon hydration. The enhanced delocalization of electrons available in HOMO as a result of the hydration of anthocyanidins can be written in the form of an order, PT > MV > DL > PO > FL > PL ≈ CY. However, no particular trend in anthocyanidins is observed in terms of orbital delocalization of LUMO. An expanded view of delocalizability in molecular orbitals lying near the frontier orbitals is given in Fig. S9. The first six unoccupied orbitals beyond HOMO, *i.e.*, LUMO, LUMO + 1, LUMO + 2, LUMO + 3, LUMO + 4 and LUMO + 5 show an increase in orbital delocalizability due to hydration for many anthocyanins. However, the five occupied molecular orbitals below HOMO did not show any specific pattern due to hydration.

### 3.3 Effect of hydration on the global descriptors

**Fig. 4:**
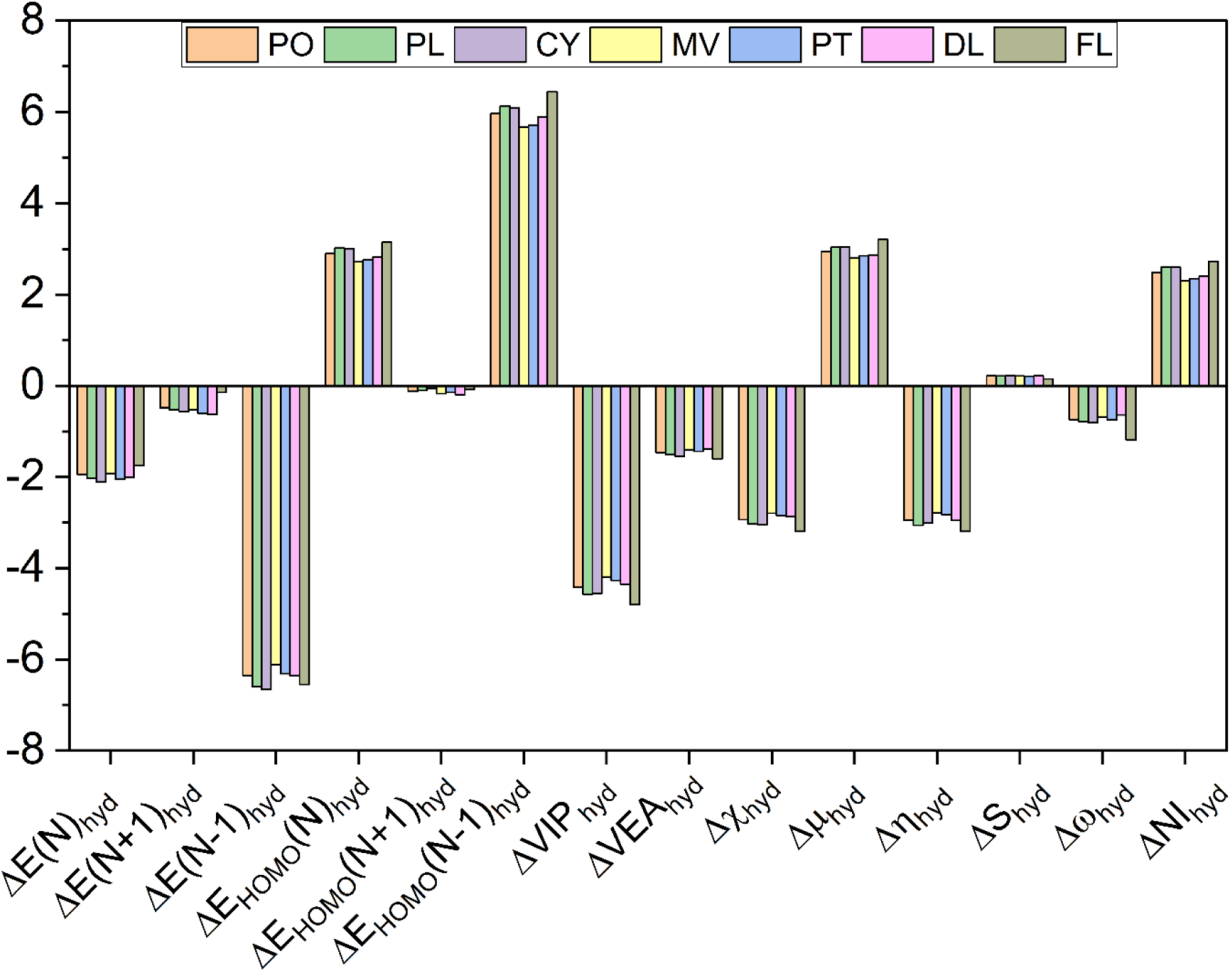
The variation of global quantum descriptors of anthocyanidins due to their hydration.

**Fig. 5:**
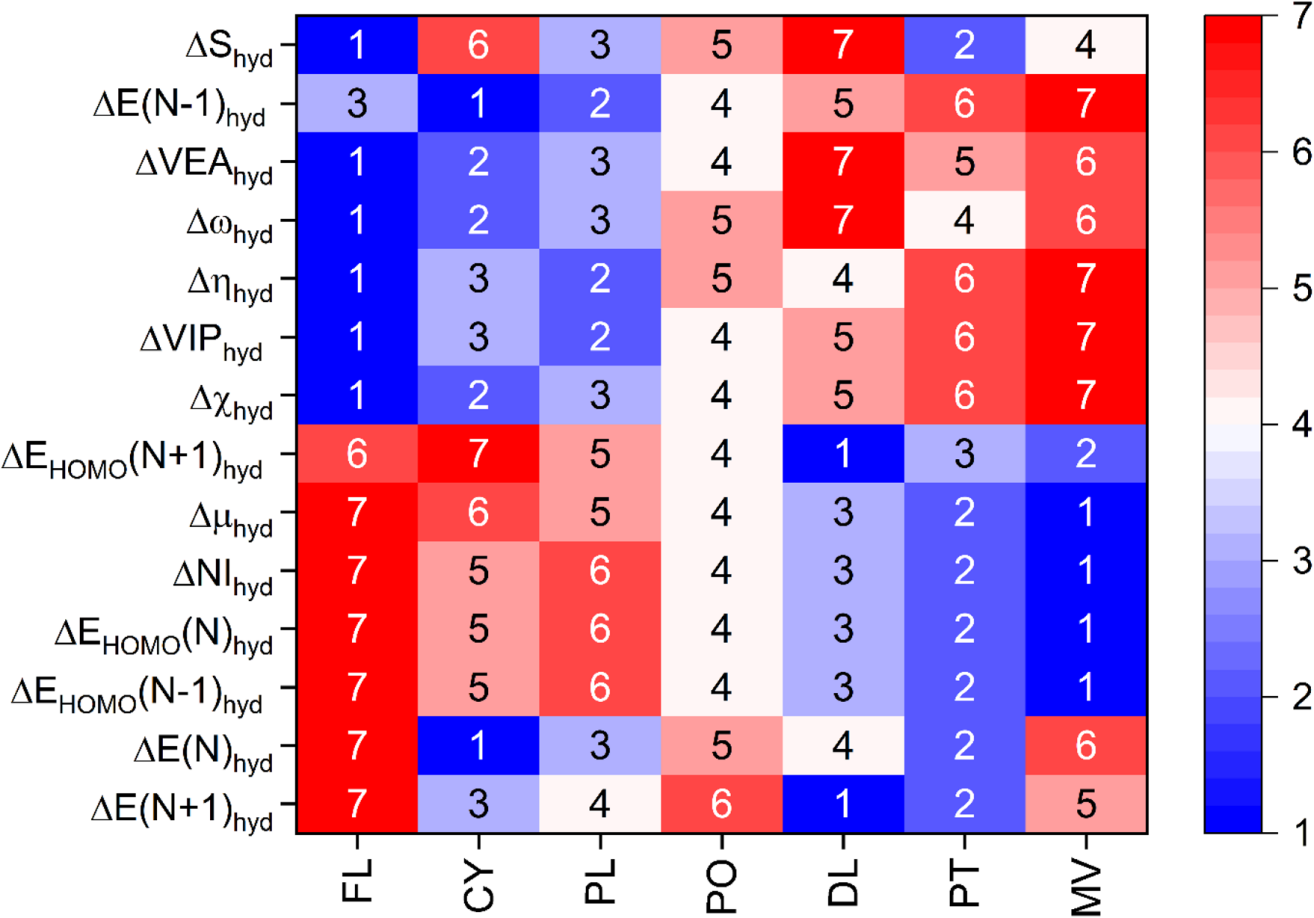
The seriated heatmap of anthocyanidins and the change in global quantum descriptors as a result of their hydration. The digits inside any particular row of the heatmap enable the column-wise ordinal ranking.

The differences between the 3D global descriptors obtained under the aqueous and gaseous phases are plotted in Fig. 4, where the descriptors have been prefixed with Δ on the abscissa of the bar chart. For this purpose, the numerical values for each of the 3D global descriptors in the gaseous phase are subtracted from the respective values in the aqueous phase. All these values are mentioned in eV for all descriptors except global chemical softness, whose value is in eV^−1^. As it is evident from the bar chart, the energy of all of the anthocyanidin molecules decreases after solvation by water, which is considered here in an implicit manner under DFT calculations. The energy of HOMO, chemical potential, global chemical softness, and nucleophilicity indices increase for all of the anthocyanidins. On the other hand, the hydration causes a decrease in the overall energy of anthocyanidin molecules, vertical ionization potential, vertical electron affinity, Mulliken electronegativity, global chemical hardness and global electrophilicity. The delocalizable positive charge on the anthocyanidin molecules under study shall be the reason for their energetic stabilization in the aqueous phase if we compare the energies of *N* + 1 and N − 1 forms with the original anthocyanidin molecules taken for the calculations. The hydration effects are causing stabilization, which is larger for the electron-deficient system (*N* − 1) than the electron-rich system (*N* + 1). For each 3D quantum descriptor, the studied anthocyanidins have been ranked according to their respective values obtained after subtraction and are shown in the form of a heatmap obtained after seriation (see Fig. 5 and Table S1). The column-wise visualization of ranks for each anthocyanidin in any particular row shall be done. The ones which have shown the smallest value of change for a particular 3D quantum descriptor upon hydration have been ranked 1, followed by others in increasing order as per their values. It should be noted that the provided ranks directly relate to values of change in the global descriptor only and not the reactivity.

The global electrophilicity of a molecule governed by *ω* provides information on the capacity to accept an arbitrary number of electrons while acting as an electrophile. The mean of global electrophilicity for anthocyanidins in gaseous and aqueous phases is 5.97 eV and 5.17 eV, respectively. These values can be used to characterize them as superelectrophiles^56^ in both phases. The anthocyanin-based films are well known to have pH dependence for their naked-eye colours. Depending on the source, aqueous solutions of anthocyanins are red or pink under acidic conditions, light pink or grey in neutral conditions and blue or green or yellow in basic conditions.^16–20^ In short, a generalization of pH-dependent colours of anthocyanins can be made as red, pink and blue in acidic, neutral and basic conditions, respectively.^21,22^ The acidic forms of anthocyanins are typically present as their final form when they are employed by mixing with polymers in the form of film.^16–20^ The presence of anthocyanin molecules enables these films for pH-sensitive applications. These films are applied as packaging films or sensors either in direct contact with food or positioned at a small distance. Both of these situations may alter the moisture content in the applied films which may affect the naked-eye colour and colorimetric sensor response of the key component of the sensor film, *i.e.*, flavylium-ion based core. The deterioration of protein content in animal-based food is known to produce amines and ammonia. The acidic form of anthocyanin molecules can sense basic species such as NH_3_ and organic amines, which are volatile in nature. Quantitatively, these basic species are often estimated as total volatile base nitrogen. The basic species may combine with moisture in the anthocyanin-based sensor film, forming its hydrated form, *e.g.*, NH_3_·H_2_O, whose hydrolysis shall yield NH_4_^+^ and OH^−^. The produced OH^−^ shall change the colour of anthocyanin molecules corresponding to their basic forms.^18^ For all anthocyanidins in both phases, the superelectrophilic character is retained and changes marginally, which is indicated by a mild decrease by a mean value of *c.a.* 0.8 eV. All anthocyanidins, except PO and PT, obtained the same rank from their Δ*ω*_hyd_ and ΔVEA_hyd_ values. For PO and PT, the ranks are interchanged between 4 and 5. From the viewpoint of electrophilicity, an order of change in the reactivity of the acidic forms of anthocyanidins with volatile nitrogenous compounds can be written as FL > CY > PL > PO ≈ PT > MV > DL. In several works, the glycone form of cyanidin is generally present along with other anthocyanin(s) in pH-sensitive applications.^17,19,20,25–31,65^ Thus, the effect of moisture on the anthocyanin-based pH-sensitive smart packaging films can be explored based on the dominant type of anthocyanin content(s) in polymer film.^22^ The trends for the ordinal ranking of anthocyanidins obtained from ΔVIP_hyd_ are quite similar to this order. The ΔNI_hyd_ exhibits a roughly opposite trend for anthocyanidins when compared with that of ΔVIP_hyd_. The mean value of the nucleophilicity index of anthocyanidins in the gaseous phase is −0.1 eV, which changes to 2.4 eV upon hydration. This change suggests that nucleophilic character increases by 2.5 eV in their hydrated forms. This observation is consistent with the prediction made by the variation in global electrophilicity values. The antioxidant properties of anthocyanins are known as they can act as reducing agent, hydrogen donor, ^1^O_2_ quencher and metal chelator.^21,39^ The free radicals and reactive oxygen species (ROS) damage packaged food and cause its spoilage, along with loss of nutrients. In general, the antioxidant activity of anthocyanins helps prevent oxidation of biomolecules, such as polyunsaturated fatty acids, proteins, and DNA.^39^ Structure–activity studies indicate that the presence of –OH groups at the C3′ and C5′ positions enhances hydrogen atom donation, implying that the ring B plays a central role in the electron-donating ability of anthocyanins.^66,67^ A higher nucleophilic character of anthocyanidins due to their hydrated forms shall enhance their antioxidant activity. Better antioxidant properties of sensor films may protect them from microbial attacks. Packaging films based on antioxidants enhance the shelf life of packaged food.^21^ It shall help them neutralize the ROS, and simultaneously, they have been shown towards their promising applications in pH-based food quality monitoring of packaged foods. For both sensing and packaging purposes, the anthocyanin-based composites also tend to reduce the oxidative stress and spoilage in the food, apart from their primary task of sensing. Their design shall be optimized to change colour in response to different levels of oxidation or spoilage inside the packaging. The analysis of DFT results shows that the reactivity of anthocyanidins towards electron-rich species is prone to be different in the gaseous and aqueous phases, even though mildly. For the situation where the anhydrous samples of anthocyanidins are being adsorbed onto a substrate or are affixed into a suitable matrix in the form of a film or strip, the colour change response by their reaction with volatile food spoilage gases (mainly organic amines and ammonia) shall be different in anhydrous and hydrated forms. The leakage of atmospheric gases may allow the moisture and aerial oxygen to penetrate the packaging, or moisture from food itself may affect the anthocyanidin-based sensors for food quality monitoring. The relative significance of hydrated and anhydrous forms of anthocyanins in sensing these species can be better evaluated through carefully controlled experiments, which may not have been comprehensively addressed in previous studies.^16–22^

The global chemical hardness gives information about the overall reactivity and stability of the molecule.^68^ A higher value of *η* indicates its resistivity towards change in its electron distribution, which is inherently stabilized.^49^ Thus, the molecule is less prone to acceptance or donation of electrons. The hydration of dried forms shall make all anthocyanidins attain lower *η* values by *c.a.* 2.9 eV (see Fig. 4). The overall increase in reactivity after hydration is predicted, and the change in reactivity order can be understood from Fig. 5. It indicates that the hydration might make anthocyanidins more susceptible towards attack by foreign species, *viz.*, superoxide radical, ammonia and so on.

The electronic chemical potential governs the ability of a system to undergo interchange of electron density with the environment at the electronic ground state. A lesser value of *µ*, which is more negative, suggests lesser stability towards the tendency to gain electrons for the studied molecule.^55^ On hydration, the value of chemical potential also increases by *c.a.* 2.97 eV. In gaseous forms, the mean value of chemical potential is −8.19 eV, while aqueous forms possess a mean chemical potential value of −5.22 eV. Thus, anthocyanidins possess strong electron-acceptor properties in dry form, which diminish to some extent on their hydration. It highlights the decreased reactivity of anthocyanidins towards above discussed foreign species in their hydrated forms, which is in line with the prediction made by other global descriptors discussed previously.

**Fig. 6:**
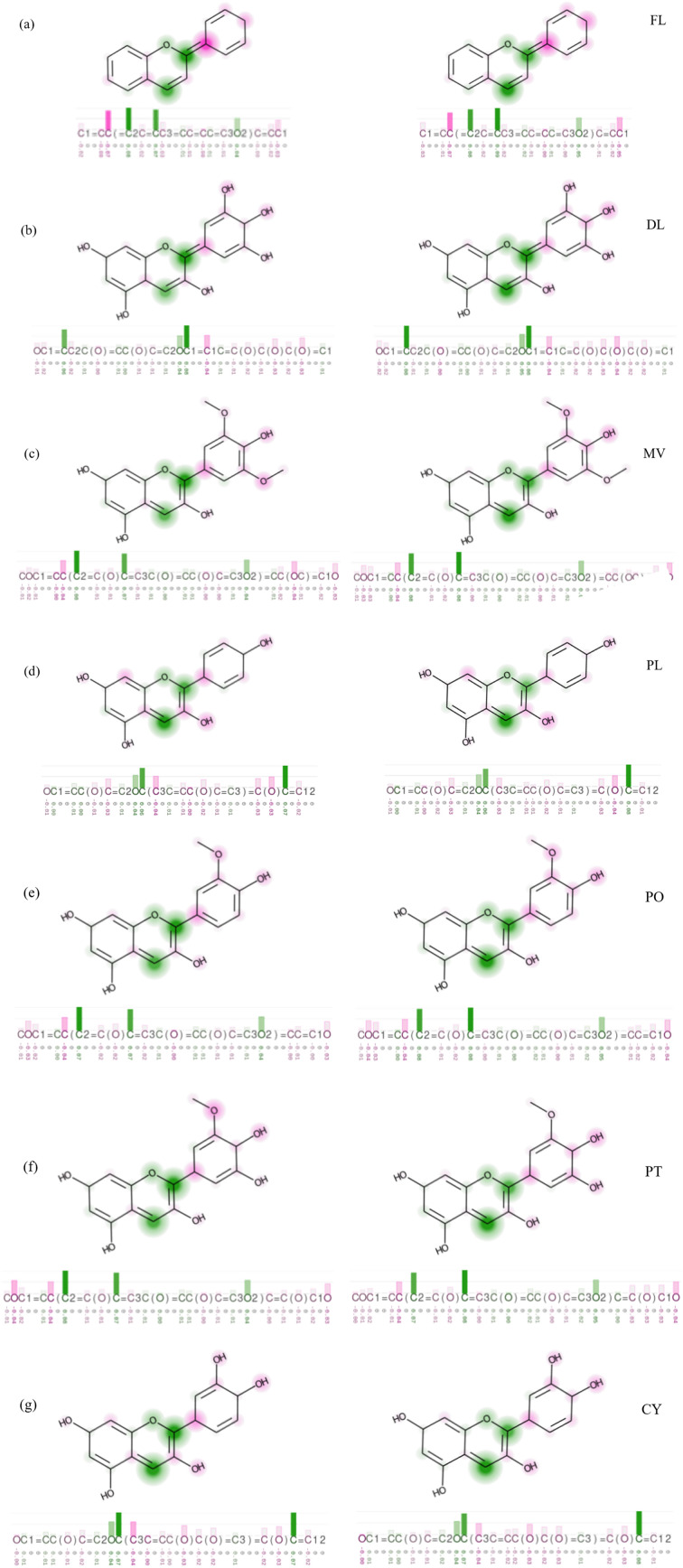
The XSMILES representation of (a) FL, (b) DL, (c) MV, (d) PL, (e) PO, (f) PT and (g) CY. The left and right portions of the diagram represent the structures with their CDD attributes in the gaseous and aqueous phases, respectively.

The global quantum descriptors offer an overall picture of a molecule's reactivity by averaging properties across the entire structure. Unlike them, the local quantum descriptors in CDFT provide detailed information about the reactivity of specific regions within a molecule. They focus mainly on the variations in electron density and electrical charges at specific atomic sites or molecular regions. This localized information is particularly beneficial in complex molecules where different regions exhibit distinct chemical behaviours. Of several local quantum descriptors known, the condensed dual descriptor^69^ (CDD) has been chosen for the visualization of reactive sites inviting attack from electron-rich and electron-deficient species. This descriptor is calculated using Hirshfeld charges of the atoms of anthocyanidins. A larger positive value of CDD suggests the site is favourable for nucleophilic attack, while its negative value suggests a favourable site for electrophilic attack, which is in accordance with previous reports.^39^ Both are of great interest in food chemistry. The visualization of CDD values is done using them as attributes for the atoms in XSMILES format representation^70^ of the anthocyanidin molecule, as shown in Fig. 6. The positive electrical charge is ignored in the canonical forms of the molecular structures. The green regions indicate positive CDD values, while the pink region indicates negative CDD values. This localized approach allows more clarity for the understanding of how different parts of a molecule contribute to its reactivity and interaction with other species. It is interesting to notice that sites for nucleophilic attack are mainly available at ring C. These sites are shown with circular green regions. Two prominent sites for nucleophilic attack are identified in ring C of anthocyanidins, *i.e.*, C2 ^71,72^ and C4 ^73^ (see Fig. 1 and 6). The oxidation of anthocyanins or their antioxidant activity is related to C2, where the addition of ROS, such as H_2_O_2_ occurs. In this oxidation, H_2_O_2_ acts as a nucleophile.^71,72^ With hydroxy and methoxy substituents on ring B, a slight alteration in the extent of electrophilicity of these sites is observed. Thus, the sites for the favourable attack by electron-rich species like ammonia and O_2_˙^−^ remain nearly the same in all cases. The height of the green bars in the bar chart over the top of the smiles string helps in visualizing the effect of solvation on these electrophilic sites. The effect of hydration on the overall reactivity of anthocyanidins can be locally visualized. In all anthocyanidins, several sites inviting the attack from electron-rich species are predicted to be more welcoming after hydration. The consideration of the hydration of anthocyanidins on the results of local and global quantum descriptors correspond well to each other and get support from orbital and electron delocalization studies.

### 3.4 Future prospects

Accumulated experimental evidences suggests that anthocyanin-based films have emerged as promising candidates for smart packaging applications due to their intrinsic pH-sensitive nature and ability to visually indicate changes in food freshness. These films, which change colour in response to environmental stimuli such as pH, are often derived from natural sources, making them suitable for sustainable packaging solutions. However, several challenges remain in translating these materials from the laboratory to real-world applications remain. Among them, hydration effects seem to pose a concern, particularly because they can significantly influence the colorimetric response, electronic structure, and reactivity of anthocyanins embedded in polymeric matrices. As demonstrated in this study, hydration induces changes in both local and global quantum descriptors of anthocyanidins, altering their aromaticity, orbital delocalization, and reactivity towards electron-rich and electron-deficient species. These theoretical insights highlight the need for precise control of moisture content in anthocyanin-based sensor films. Therefore, future experimental strategies should prioritize controlling the hydration state of these films during storage, application, and usage to maintain their sensitivity and reliability in detecting volatile amines or ammonia released during food spoilage.

Another critical consideration is the integration of these films into packaging systems. While direct contact with food is a common practice, it may not always be optimal, especially if the food is moist or emits water vapor. To mitigate this, it may be advantageous to design packaging systems where the colorimetric indicator label is placed at a slight distance from the food, potentially within a semi-permeable window that allows volatile gases to reach the sensor without excessive moisture ingress. Such design modifications can help maintain the sensor's dry or partially hydrated state, in an attempt towards optimizing its performance.

Moreover, environmental variables such as ionic strength, storage temperature, and relative humidity must be accounted for, as they could further influence hydration levels and, consequently, the stability and optical behaviour of anthocyanin films. The engineering of smart packaging materials, therefore, should focus on material compatibility and barrier properties, sensor placement, and film encapsulation techniques that minimize undesirable hydration while allowing for analyte diffusion.

The advances in polymer engineering, including the development of hydrophobic or water-regulating barrier layers, could provide solutions to modulate the hydration kinetics of anthocyanin films. Future work should also explore cross-linking strategies or composite film formulations that stabilize anthocyanins in desired protonation states while preserving their responsiveness. Coating anthocyanin films with biocompatible nanolayers or integrating them into multi-layer smart packaging systems may further enhance stability against humidity while preserving colorimetric sensitivity.

The increasing use of smartphone-based imaging and analysis tools for colorimetric interpretation introduces another layer of variability while having its own advantages. Future research may also involve machine learning-assisted calibration models that account for hydration and other factor-dependent shifts in colour profiles, thereby improving the accuracy and robustness of food quality assessments under real-world conditions. Collectively, the insights from this study lay a foundation for the rational design of hydration-tolerant, pH-sensitive smart packaging systems, paving the way for more reliable and scalable applications in food monitoring and preservation.

## 4 Conclusion

The aromaticity, electronic structure, and global reactivity of anthocyanidins are studied in both gaseous and aqueous phases using the Harmonic Oscillator Model of Aromaticity (HOMA), *para*-delocalization index (PDI), and orbital delocalization index (ODI), alongside conceptual density functional theory (CDFT). The key findings indicate that hydration influences the aromaticity of anthocyanidins differently in their various six-membered rings. The HOMA and PDI analyses reveal that ring B exhibits the highest aromaticity, followed by ring A and ring C. The hydration generally increases the aromaticity of all rings, though the extent of the increase varies between the indices. PDI consistently shows that ring B maintains superior aromaticity compared to rings A and C, both before and after hydration. HOMA indices also suggest that the aromaticity of ring C improves more significantly upon hydration, reflecting a different approach in assessing aromaticity as compared to PDI. Further analysis using ODI indicates that hydration enhances the delocalization of electrons in the HOMO of anthocyanidins, although no significant trend is observed for the LUMO. This enhanced delocalization correlates with the increased reactivity of anthocyanidins in their hydrated forms as predicted from local quantum descriptors. The condensed dual descriptors show the enhancement in local reactivity of some specific sites due to hydration. The global reactivity descriptors calculated *via* CDFT reveal that hydration generally lowers the global chemical hardness and increases the chemical softness, chemical potential, and nucleophilicity of anthocyanidins. They collectively suggest that hydrated anthocyanidins are slightly less reactive but remain susceptible to interactions with ammoniacal compounds and other species. The study shows how hydration affects the reactivity of anthocyanidins, providing quantum-level insights that may assist in developing predictive models for designing anthocyanin-based materials in food preservation and shelf-life monitoring. While primarily theoretical, these findings can guide future experimental and computational efforts toward optimizing anthocyanin stability with moisture dependent functionality in real-world applications.

## Conflicts of interest

The author declares no competing financial interests or conflicts of interest.

## Supplementary Material

RA-016-D5RA05334J-s001

## Data Availability

The data is available on request. Supplementary information (SI) is available. See DOI: https://doi.org/10.1039/d5ra05334j.

## References

[cit1] Sendri N., Bhandari P. (2024). Phytochem. Rev..

[cit2] Amić D., Davidović-Amić D., Bešlo D., Lučić B., Trinajstić N. (1999). J. Chem. Inf. Comput. Sci..

[cit3] Qin B., Anderson R. A. (2012). Br. J. Nutr..

[cit4] Kong J.-M., Chia L.-S., Goh N.-K., Chia T.-F., Brouillard R. (2003). Phytochemistry.

[cit5] Krga I., Milenkovic D. (2019). J. Agric. Food Chem..

[cit6] Jiang T., Shuai X., Li J., Yang N., Deng L., Li S., He Y., Guo H., Yubao L., He J. (2020). J. Agric. Food Chem..

[cit7] Mei L. X., Nafchi A. M., Ghasemipour F., Easa A. M., Jafarzadeh S., Al-Hassan A. A. (2020). Int. J. Biol. Macromol..

[cit8] Dong M., He X., Liu R. H. (2007). J. Agric. Food Chem..

[cit9] Sanches M. A. R., Camelo-Silva C., Carvalho C. da S., de Mello J. R., Barroso N. G., Barros E. L. da S., Silva P. P., Pertuzatti P. B. (2021). LWT.

[cit10] Luchese C. L., Uranga J., Spada J. C., Tessaro I. C., de la Caba K. (2018). Int. J. Biol. Macromol..

[cit11] Ge Y., Li Y., Bai Y., Yuan C., Wu C., Hu Y. (2020). Int. J. Biol. Macromol..

[cit12] Zhang X., Liu Y., Yong H., Qin Y., Liu J., Liu J. (2019). Food Hydrocoll..

[cit13] Zhai X., Shi J., Zou X., Wang S., Jiang C., Zhang J., Huang X., Zhang W., Holmes M. (2017). Food Hydrocoll..

[cit14] Goodarzi M. M., Moradi M., Tajik H., Forough M., Ezati P., Kuswandi B. (2020). Int. J. Biol. Macromol..

[cit15] Moradi M., Tajik H., Almasi H., Forough M., Ezati P. (2019). Carbohydr. Polym..

[cit16] Gasti T., Dixit S., D’souza O. J., Hiremani V. D., Vootla S. K., Masti S. P., Chougale R. B., Malabadi R. B. (2021). Int. J. Biol. Macromol..

[cit17] Zhang J., Zou X., Zhai X., Huang X., Jiang C., Holmes M. (2019). Food Chem..

[cit18] Jiang G., Hou X., Zeng X., Zhang C., Wu H., Shen G., Li S., Luo Q., Li M., Liu X., Chen A., Wang Z., Zhang Z. (2020). Int. J. Biol. Macromol..

[cit19] Zhang K., Huang T.-S., Yan H., Hu X., Ren T. (2020). Int. J. Biol. Macromol..

[cit20] Zeng P., Chen X., Qin Y.-R., Zhang Y.-H., Wang X.-P., Wang J.-Y., Ning Z.-X., Ruan Q.-J., Zhang Y.-S. (2019). Food Res. Int..

[cit21] Yong H., Liu J. (2020). Food Packag. Shelf Life.

[cit22] Zhao L., Liu Y., Zhao L., Wang Y., Agric J. (2022). Food Res..

[cit23] Hohenberg P., Kohn W. (1964). Phys. Rev..

[cit24] Kohn W., Sham L. J. (1965). Phys. Rev..

[cit25] Yang M., Lin J., Zhang M., Zhuang Y., Li Y., Wang B., Zhang Z., Liu J., Fei P. (2025). Food Chem..

[cit26] Xu M., Fang D., Kimatu B. M., Lyu L., Wu W., Cao F., Li W. (2024). Food Control.

[cit27] Xiao N., Chen R., Xiang H., He J., Li S., Chen X., Zhu Z. (2025). Food Packag. Shelf Life.

[cit28] Huang J.-Y., Chen Y.-L., Lin D.-Q., Sun L.-C., Liu K., Zhang L.-J., Hu Y.-Q., Cao M.-J. (2025). Food Chem..

[cit29] Amaregouda Y., Kamanna K. (2023). Sustainable Food Technol..

[cit30] Sharma C., Kundu S., Singh S., Saxena J., Gautam S., Kumar A., Pathak P. (2025). RSC Sustainability.

[cit31] Palanisamy Y., Kadirvel V., Ganesan N. D. (2025). Sustainable Food Technol..

[cit32] Wallace T. C. and Giusti M. M., Anthocyanins in Health and Disease, CRC Press, Taylor & Francis Group, 2014, 10.1201/b15554

[cit33] Tang B., Li L., Hu Z., Chen Y., Tan T., Jia Y., Xie Q., Chen G. (2020). J. Agric. Food Chem..

[cit34] Horiuchi R., Nishizaki Y., Okawa N., Ogino A., Sasaki N. (2020). J. Agric. Food Chem..

[cit35] Vo T.-V., Dang T.-H., Chen B.-H. (2019). Polymers.

[cit36] Goudarzi J., Moshtaghi H., Shahbazi Y. (2023). Food Packag. Shelf Life.

[cit37] Schleyer P. V. R., Maerker C., Dransfeld A., Jiao H., Van Eikema Hommes N. J. R. (1996). J. Am. Chem. Soc..

[cit38] Fallah-Bagher-Shaidaei H., Wannere C. S., Corminboeuf C., Puchta R., Schleyer P. V. R. (2006). Org. Lett..

[cit39] Dangles O., Fenger J.-A. (2018). Molecules.

[cit40] Krishnan R., Binkley J. S., Seeger R., Pople J. A. (1980). J. Chem. Phys..

[cit41] Lee C., Yang W., Parr R. G. (1988). Phys. Rev. B: Condens. Matter Mater. Phys..

[cit42] Becke A. D. (1993). J. Chem. Phys..

[cit43] Stephens P. J., Devlin F. J., Chabalowski C. F., Frisch M. J. (1994). J. Phys. Chem..

[cit44] Luo Y.-C., Jing P. (2021). Food Chem.: Mol. Sci..

[cit45] Liu S., Ma C., Zhang Y., Wang Y., Tian J., Li B., Zhao J. (2024). eFood.

[cit46] Grimme S., Ehrlich S., Goerigk L. (2011). J. Comput. Chem..

[cit47] Klamt A., Moya C., Palomar J. (2015). J. Chem. Theory Comput..

[cit48] Frisch M. J. , Trucks G. W., Schlegel H. B., Scuseria G. E., Robb M. A., Cheeseman J. R., Scalmani G., Barone V., Petersson G. A., Nakatsuji H., Li X., Caricato M., Marenich A. V., Bloino J., Janesko B. G., Gomperts R., Mennucci B., Hratchian H. P., Ortiz J. V., Izmaylov A. F., Sonnenberg J. L., Williams-Young D., Ding F., Lipparini F., Egidi F., Goings J., Peng B., Petrone A., Henderson T., Ranasinghe D., Zakrzewski V. G., Gao J., Rega N., Zheng G., Liang W., Hada M., Ehara M., Toyota K., Fukuda R., Hasegawa J., Ishida M., Nakajima T., Honda Y., Kitao O., Nakai H., Vreven T., Throssell K., Montgomery Jr J. A., Peralta J. E., Ogliaro F., Bearpark M. J., Heyd J. J., Brothers E. N., Kudin K. N., Staroverov V. N., Keith T. A., Kobayashi R., Normand J., Raghavachari K., Rendell A. P., Burant J. C., Iyengar S. S., Tomasi J., Cossi M., Millam J. M., Klene M., Adamo C., Cammi R., Ochterski J. W., Martin R. L., Morokuma K., Farkas O., Foresman J. B. and Fox D. J., Gaussian 16 Revision B.01, Gaussian, Inc., Wallingford CT

[cit49] Chermette H. (1999). J. Comput. Chem..

[cit50] Lu T., Chen F. (2012). J. Comput. Chem..

[cit51] Parr R. G., Pearson R. G. (1983). J. Am. Chem. Soc..

[cit52] Parr R. G., Donnelly R. A., Levy M., Palke W. E. (1978). J. Chem. Phys..

[cit53] Parr R. G., Szentpály L. V., Liu S. (1999). J. Am. Chem. Soc..

[cit54] Domingo L. R., Chamorro E., Pérez P. (2008). J. Org. Chem..

[cit55] Domingo L., Ríos-Gutiérrez M., Pérez P. (2016). Molecules.

[cit56] Ríos-Gutiérrez M., Saz Sousa A., Domingo L. R. (2023). J. Phys. Org. Chem..

[cit57] Shankar A. (2025). Results Chem..

[cit58] Iraqui S., Shankar A., Dubey A., Jaiswal A. (2025). J. Environ. Manage..

[cit59] Kruszewski J., Krygowski T. M. (1972). Tetrahedron Lett..

[cit60] Krygowski T. M. (1993). J. Chem. Inf. Comput. Sci..

[cit61] Poater J., Fradera X., Duran M., Solà M. (2003). Chem.–Eur. J..

[cit62] Bader R. F. W. (1985). Acc. Chem. Res..

[cit63] Bader R. F. W. (1991). Chem. Rev..

[cit64] Lu T., Chen F. (2011). Acta Chim. Sin..

[cit65] León-Vázquez B. B., Rodríguez-Félix F., Torres-Arreola W., Aubourg S. P., Graciano-Verdugo A. Z., Plascencia-Jatomea M., Quintero-Reyes I. E., Urías-Torres M. Á., Moreno-Robles A. L., Tapia-Hernández J. A., Moreno-Vásquez M. J. (2025). Int. J. Biol. Macromol..

[cit66] Kähkönen M. P., Heinonen M. (2003). J. Agric. Food Chem..

[cit67] Sadowska-Bartosz I., Bartosz G. (2024). Int. J. Mol. Sci..

[cit68] Pearson R. G. (1986). Proc. Natl. Acad. Sci. U. S. A..

[cit69] Morell C., Grand A., Toro-Labbé A. (2005). J. Phys. Chem. A.

[cit70] Heberle H., Zhao L., Schmidt S., Wolf T., Heinrich J. (2023). J. Cheminf..

[cit71] Dueñas M., Fulcrand H., Cheynier V. (2006). Anal. Chim. Acta.

[cit72] Lopes P., Richard T., Saucier C., Teissedre P.-L., Monti J.-P., Glories Y. (2007). J. Agric. Food Chem..

[cit73] Berké B., Chèze C., Vercauteren J., Deffieux G. (1998). Tetrahedron Lett..

