# Supplementary material for: Hydration-induced modulation of aromaticity and reactivity in anthocyanidins: a quantum mechanical study
Source: RSC Adv. 2026 Jan 22;16(5):4461–72. doi: 10.1039/d5ra05334j (PMC12825114; doi:10.1039/d5ra05334j)
Supplement: RA-016-D5RA05334J-s001 [file RA-016-D5RA05334J-s001.pdf]

Supplementary Information (SI) for RSC Advances.
This journal is © The Royal Society of Chemistry 2026

Supplementary Information

Hydration-induced modulation of aromaticity and reactivity in anthocyanidins: a quantum mechanical study

Ajay Shankar*

Department of Chemistry, Indira Gandhi National Tribal University, Amarkantak, Madhya Pradesh – 484887, India.
*Email: ashankar@igntu.ac.in

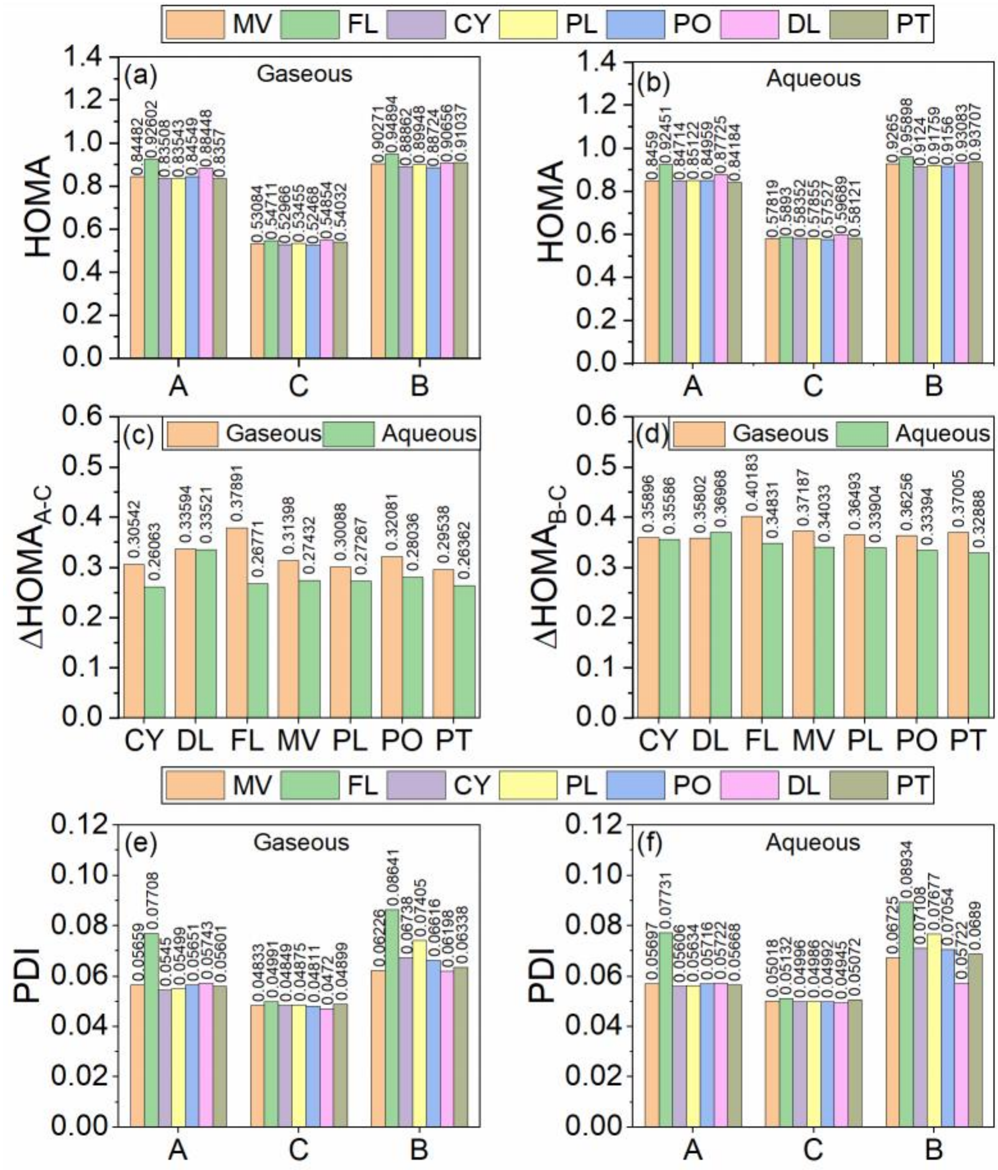

Figure S1. HOMA indices of the three rings of anthocyanidins in (a) gaseous phase and (b) aqueous phase. The difference between HOMA indices of (c) fused rings (d) ring B and ring C. The PDI values of the three rings of anthocyanidins in (d) gaseous phase and (e) aqueous phase.

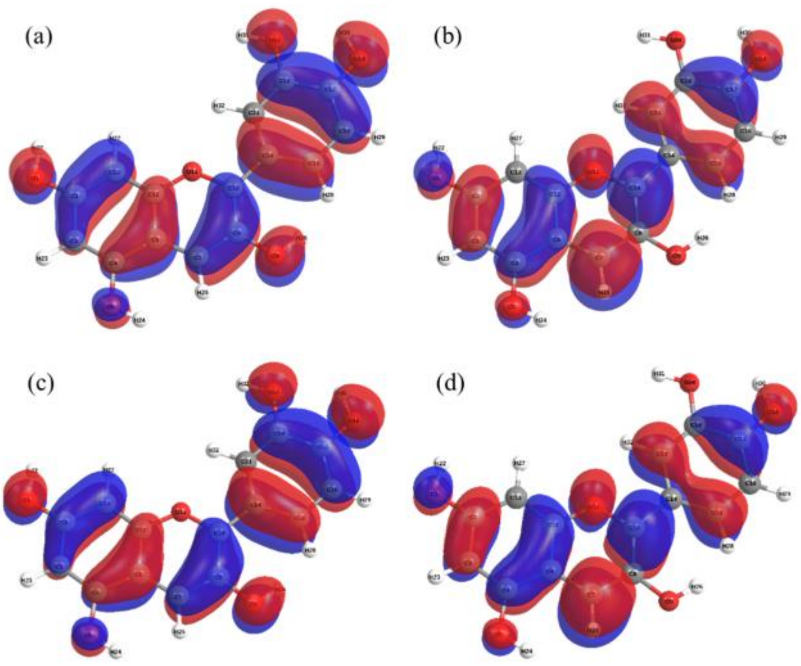

Figure S2. The isosurface plots of HOMO and LUMO for CY in different phases: (a) HOMO in the gaseous phase, (b) LUMO in the gaseous phase, (c) HOMO in the aqueous phase, and (d) LUMO in the aqueous phase.

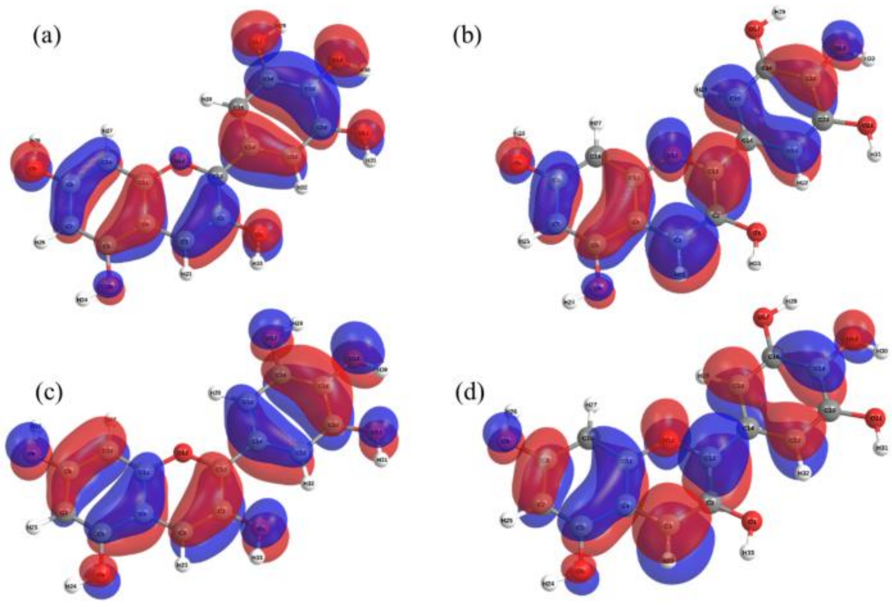

Figure S3. The isosurface plots of HOMO and LUMO for DL in different phases: (a) HOMO in the gaseous phase, (b) LUMO in the gaseous phase, (c) HOMO in the aqueous phase, and (d) LUMO in the aqueous phase.

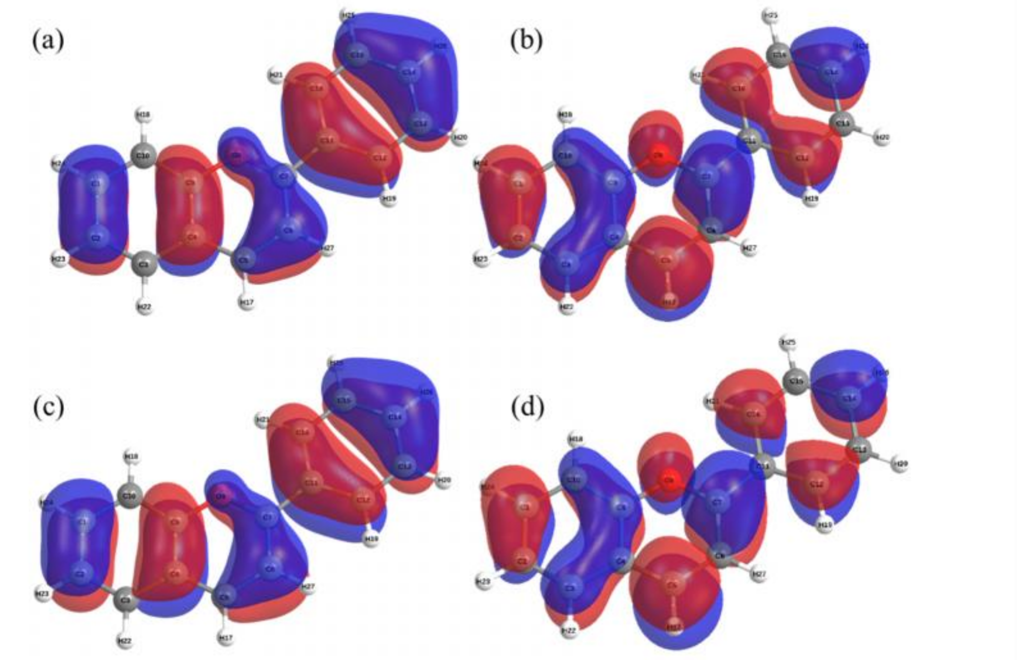

Figure S4. The isosurface plots of HOMO and LUMO for FL in different phases: (a) HOMO in the gaseous phase, (b) LUMO in the gaseous phase, (c) HOMO in the aqueous phase, and (d) LUMO in the aqueous phase.

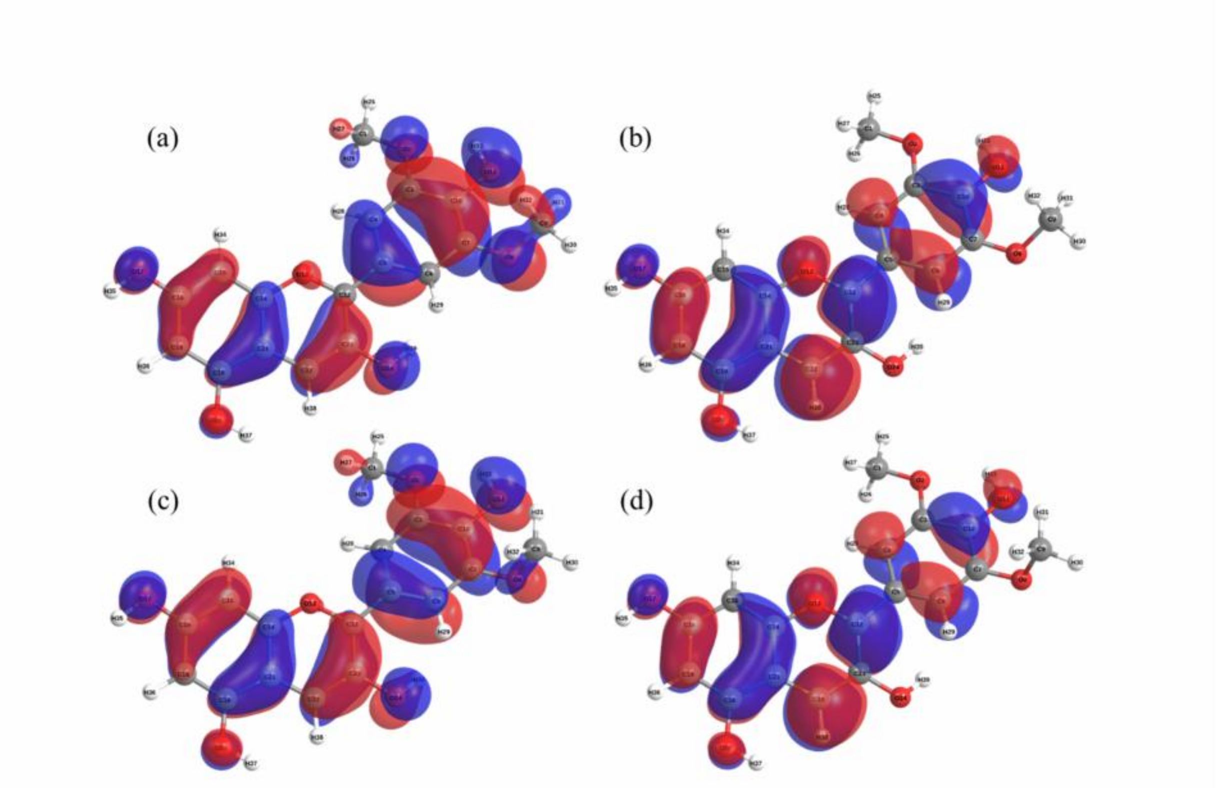

Figure S5. The isosurface plots of HOMO and LUMO for MV in different phases: (a) HOMO in the gaseous phase, (b) LUMO in the gaseous phase, (c) HOMO in the aqueous phase, and (d) LUMO in the aqueous phase.

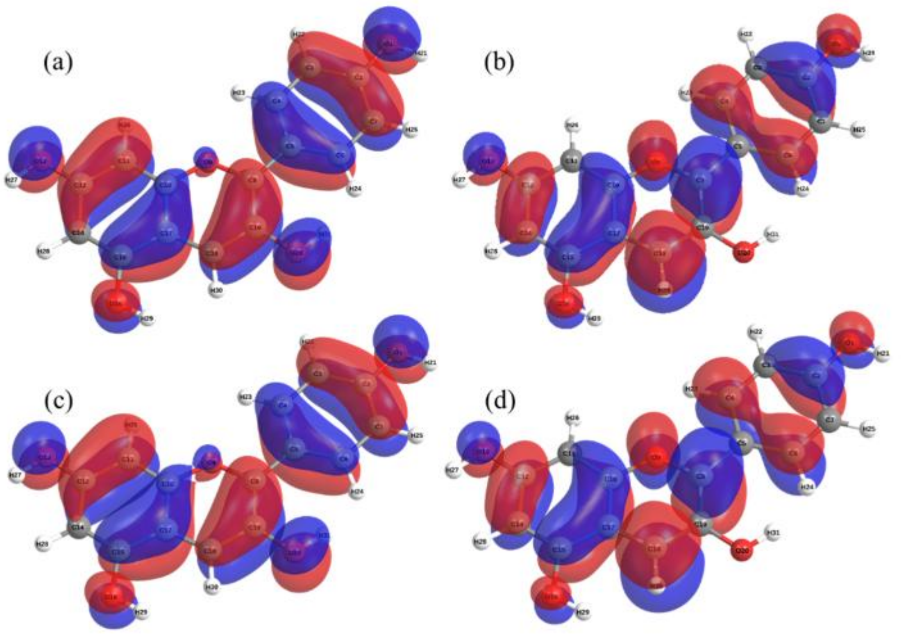

Figure S6. The isosurface plots of HOMO and LUMO for PL in different phases: (a) HOMO in the gaseous phase, (b) LUMO in the gaseous phase, (c) HOMO in the aqueous phase, and (d) LUMO in the aqueous phase.

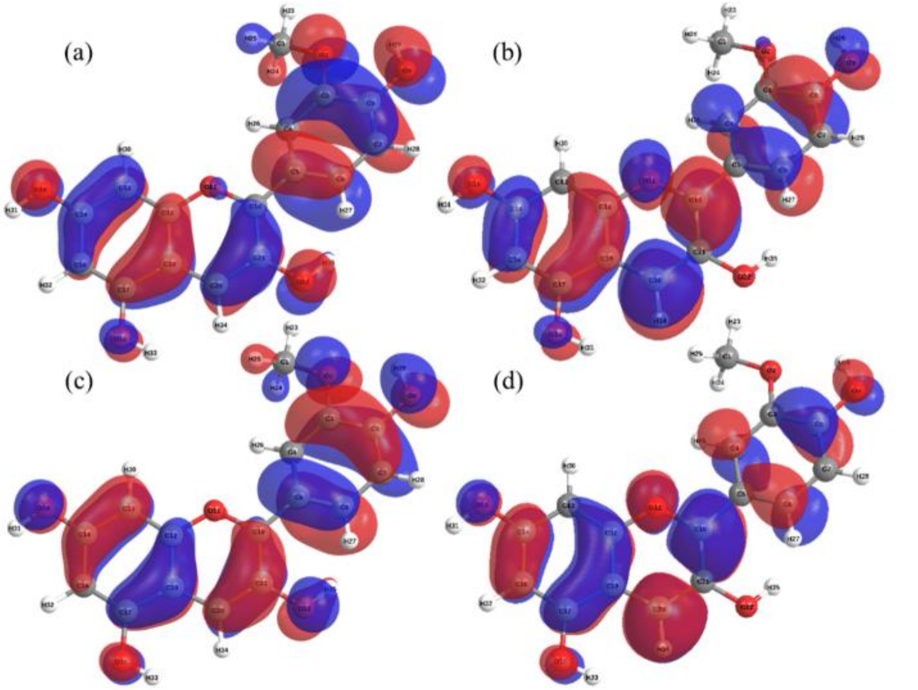

Figure S7. The isosurface plots of HOMO and LUMO for PO in different phases: (a) HOMO in the gaseous phase, (b) LUMO in the gaseous phase, (c) HOMO in the aqueous phase, and (d) LUMO in the aqueous phase.

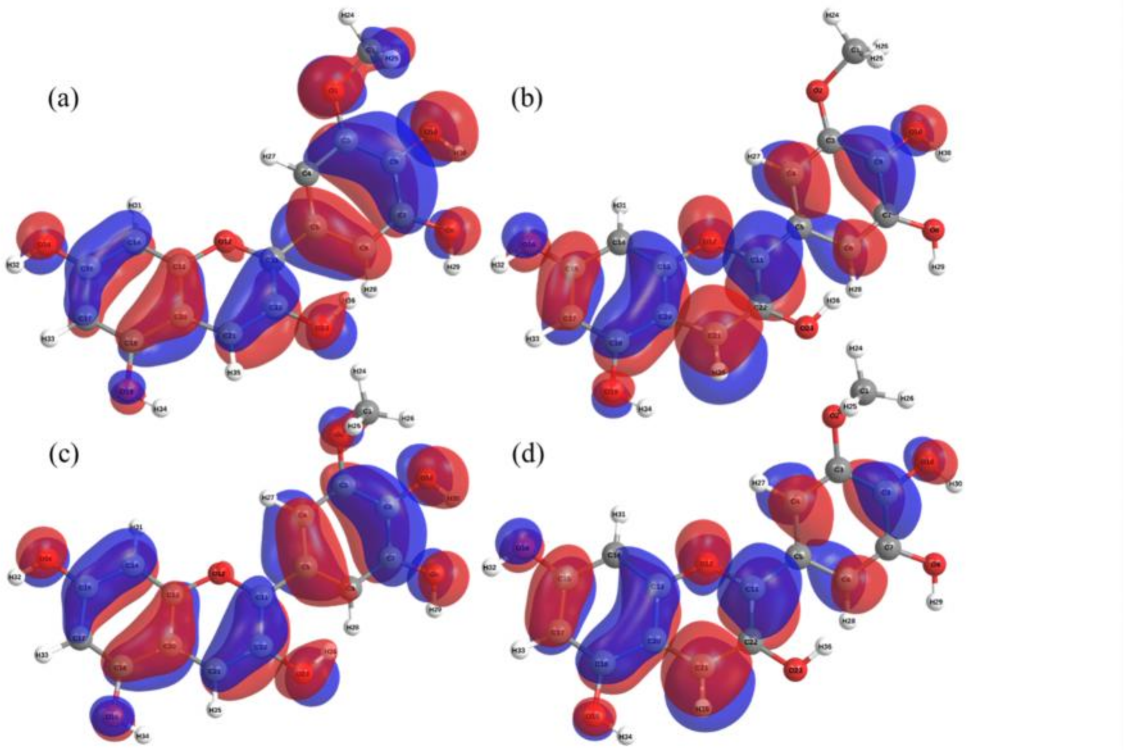

Figure S8. The isosurface plots of HOMO and LUMO for PT in different phases: (a) HOMO in the gaseous phase, (b) LUMO in the gaseous phase, (c) HOMO in the aqueous phase, and (d) LUMO in the aqueous phase.

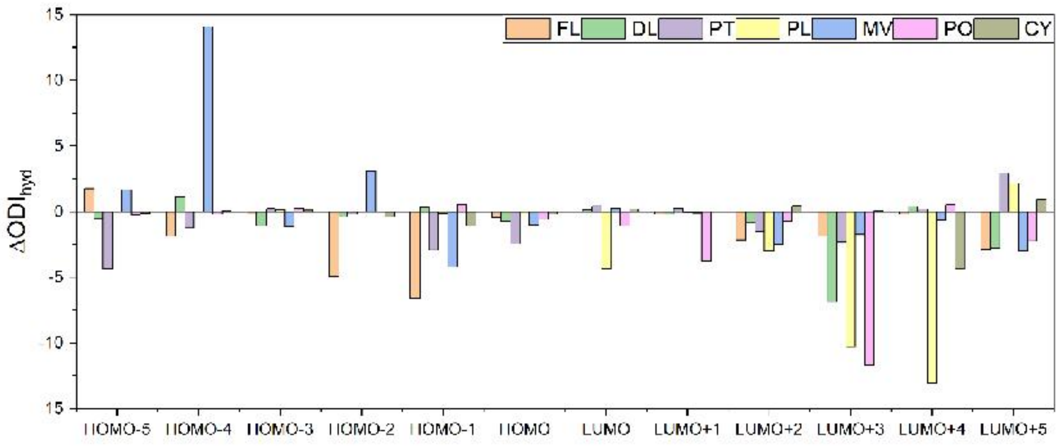

Figure S9. The effect of hydration on ODI values of 5 molecular orbitals below and above frontier orbitals.

Table S1. The change in global quantum descriptors of anthocyanidins as obtained from CDFT.

	CY	DL	FL	MV	PL	PO	PT
$\Delta E(N)_{hyd}$	-2.1084	-2.0111	-1.7507	-1.9213	-2.0312	-1.9465	-2.0425
$\Delta E(N+1)_{hyd}$	-0.5679	-0.6203	-0.1521	-0.5198	-0.5260	-0.4801	-0.6050
$\Delta E(N-1)_{hyd}$	-6.6622	-6.3631	-6.5509	-6.1223	-6.6002	-6.3632	-6.3127
$\Delta E_{HOMO}(N)_{hyd}$	3.0109	2.8211	3.1402	2.7143	3.0180	2.8957	2.7594
$\Delta E_{HOMO}(N+1)_{hyd}$	-0.0623	-0.1998	-0.0853	-0.1731	-0.1087	-0.1269	-0.1527
$\Delta E_{HOMO}(N-1)_{hyd}$	6.0912	5.8977	6.4421	5.6659	6.1345	5.9643	5.7149
ΔVIP_{hyd}	-4.5537	-4.3522	-4.8002	-4.2010	-4.5690	-4.4167	-4.2702
ΔVEA_{hyd}	-1.5405	-1.3907	-1.5985	-1.4015	-1.5052	-1.4663	-1.4375
$\Delta \chi_{hyd}$	-3.0471	-2.8714	-3.1994	-2.8012	-3.0372	-2.9415	-2.8538
$\Delta \mu_{hyd}$	3.0471	2.8714	3.1994	2.8012	3.0372	2.9415	2.8538
$\Delta \eta_{hyd}$	-3.0132	-2.9614	-3.2017	-2.7995	-3.0639	-2.9504	-2.8326
ΔS_{hyd}	0.2154	0.2163	0.1472	0.2097	0.2084	0.2129	0.2052
$\Delta \omega_{hyd}$	-0.8051	-0.6356	-1.1853	-0.6809	-0.7887	-0.7442	-0.7461
ΔNI_{hyd}	2.5994	2.4096	2.7287	2.3028	2.6065	2.4842	2.3479
